# Hybrid RNA Sequencing Strategy for the Dynamic Transcriptomes of Winter Dormancy in an Evergreen Herbaceous Perennial, *Iris japonica*

**DOI:** 10.3389/fgene.2022.841957

**Published:** 2022-03-16

**Authors:** Danqing Li, Lingmei Shao, Tong Xu, Xiaobin Wang, Runlong Zhang, Kaijing Zhang, Yiping Xia, Jiaping Zhang

**Affiliations:** Genomics and Genetic Engineering Laboratory of Ornamental Plants, College of Agriculture and Biotechnology, Zhejiang University, Hangzhou, China

**Keywords:** *Iris japonica*, evergreen, hybrid sequencing, single-molecule real-time sequencing, gene expression, real-time qPCR

## Abstract

Japanese iris (*Iris japonica*) is a popular perennial ornamental that originated in China; it has a long display period and remains green outdoors throughout the year. winter dormancy characteristics contribute greatly to the evergreenness of herbaceous perennials. Thus, it is crucial to explore the mechanism of winter dormancy in this evergreen herbaceous perennial. Here, we used the hybrid RNA-seq strategy including single-molecule real-time (SMRT) and next-generation sequencing (NGS) technologies to generate large-scale Full-length transcripts to examine the shoot apical meristems of Japanese iris. A total of 10.57 Gb clean data for SMRT and over 142 Gb clean data for NGS were generated. Using hybrid error correction, 58,654 full-length transcripts were acquired and comprehensively analysed, and their expression levels were validated by real-time qPCR. This is the first full-length RNA-seq study in the *Iris* genus; our results provide a valuable resource and improve understanding of RNA processing in this genus, for which little genomic information is available as yet. In addition, our data will facilitate in-depth analyses of winter dormancy mechanisms in herbaceous perennials, especially evergreen monocotyledons.

## Introduction

Evergreen ornamentals are desirable for landscapes and gardens, as they not only have a prolonged display duration but also provide scarce greenery in the winter (Guo et al., 2012; Li et al., 2017; Li et al., 2019). Previous studies have indicated that the growth-to-dormancy transition might play an essential role in the winter performance of evergreen herbaceous perennials (Adams et al., 2004; Azimi et al., 2016; Li et al., 2021). Moreover, winter dormancy could contribute greatly to plant growth adaptability in unfavourable environments (Azizi Gannouni et al., 2017; Gillespie and Volaire, 2017; Beauvieux et al., 2018), blooming and fruit bearing in the next year and a series of developmental processes (Castede et al., 2015; Saito et al., 2015; Wu et al., 2017). In general, deciduous trees have the capacity to time their periods of dormancy accurately by detecting a critical day length, and then, decreasing temperatures result in enhanced cold tolerance and leaf loss (Arora et al., 2003; Fennell et al., 2015; Zohner and Renner, 2015; Maurya and Bhalerao, 2017). This process involves a complex regulatory network in the terminal buds, including physiological, biochemical, transcriptional and epigenetic regulation (Falavigna et al., 2019; Wu et al., 2019). Although great progress has been made in the study of the mechanism of winter dormancy in deciduous woody plants (Chao et al., 2017; Tylewicz et al., 2018; Singh et al., 2019), this process remains unclear in herbaceous perennials, especially evergreen ones.

*Iris* L. is one of the most horticulturally important plants and has high commercial value all over the world (Azimi et al., 2016; Li et al., 2016; Wang et al., 2018). At present, over 70,000 iris cultivars with striking ornamental traits and growth adaptability are bred worldwide (Hu and Xiao, 2012; Ruan et al., 2017; Azimi et al., 2018; Fan et al., 2020). Interestingly, there are significant differences in the foliar habits of irises, which can be classified as evergreen, semi-evergreen and deciduous (Streich and Steinegger, 2000). Therefore, irises are an appropriate subject to study the winter dormancy traits of evergreen herbaceous perennials. To address our research questions, we selected Japanese iris (*I. japonica*), one of the most broadly used evergreen iris species in landscaping and gardens, which originated and is cultivated widely in China, as an ideal material for exploring the mechanism of winter dormancy in evergreen herbaceous perennials. However, due to the large genome sizes (∼10 Gb) of irises (Kentner et al., 2003), genomic information in the *Iris* genus is not available. Thus, it is difficult to understand the underlying mechanism of winter dormancy in Japanese iris at the molecular level.

Transcriptome sequencing could provide comprehensive and accurate information on gene expression in specific developmental processes or tissues of an organism (Ozsolak and Milos, 2011; Romero et al., 2012). Due to the decreasing cost of next-generation sequencing (NGS) technologies, many key genes related to the regulation of perennial winter dormancy, such as *DORMANCY ASSOCIATED MADS-BOX* (*DAM*), *SUPPRESSOR OF OVEREXPRESSION OF CONSTANS 1* (*SOC1*), *FLOWERING LOCUS T* (*FT*) and *FLOWERING LOCUS F* (*FLC*), have been identified in perennial plants (Shim et al., 2014; Howe et al., 2015; Voogd et al., 2015). Moreover, the gene network responsible for regulation of the stress response has been elucidated in the *Iris* genus by studying the transcript profiles of different tissues under different treatments using NGS (Tian et al., 2015; Gu et al., 2017; Gu et al., 2018; Liu et al., 2018). Nevertheless, full-length (FL) RNA-seq datasets are currently unavailable in the *Iris* genus. The relatively short read lengths generated by NGS prevent the accurate assembly of FL transcripts. In addition, the low quality of the transcripts assembled from short-read RNA-seq reduces the accuracy of annotations (Olasz et al., 2020; Tombacz et al., 2021).

Single-molecule real-time (SMRT) sequencing, a third-generation sequencing technology, could eliminate the need for assembly with much longer reads and provide direct evidence for the transcript isoforms of each gene (McCarthy, 2010; Tilgner et al., 2014; Minoche et al., 2015). Furthermore, the higher error rate associated with SMRT sequencing has been addressed by hybrid error correction using NGS (Koren et al., 2012). The hybrid strategy to combine SMRT and NGS for RNA-seq could take advantage of both sequencing technologies, without computational assembly of Illumina short reads and avoiding the limitations of long reads with higher error rates and low throughput. At present, this hybrid sequencing strategy has been increasingly applied in genome annotation, new gene discovery and transcriptomic research (Wang K. et al., 2019; Wang X. et al., 2019; Zhang et al., 2019; Wang et al., 2021). Therefore, this combined RNA-seq technology could provide important support for in-depth research on winter dormancy in evergreen herbaceous perennials, especially when genomic information is still unavailable.

Herein, we used the hybrid “SMRT + NGS” RNA-seq strategy to generate large-scale FL transcripts and generate a gene expression profile of winter dormancy in Japanese iris. Additionally, quality control was performed to evaluate the quality of the RNA samples, sequencing, gene annotation, structure and expression, and a high-quality dataset is presented. These FL transcripts will provide gene sequence information for the further study of irises, and the gene expression profiles will provide a comprehensive understanding of the winter dormancy and foliar habits of herbaceous perennials, especially evergreen monocotyledons.

## Materials and Methods

### Plant Materials

One-year-old uniformly sized Japanese iris plants were potted without shelters at the Resource Nursery for Flower Bulbs and Herbaceous Perennials, Zhejiang University, Hangzhou (N, 29° 11′ - 30° 33′; E, 118° 21′ - 120° 30′), China. Apical shoot samples (including the shoot apex and the three youngest leaves) of different developmental stages in the natural environment were collected every 2 weeks beginning on 13 November 2015. The samples were transferred to liquid nitrogen immediately and stored at −80°C. Simultaneously, the growth or dormancy status of the plants was observed during sampling. The samples were designated Ever 1 to Ever 10 based on the order of sampling.

### RNA Preparation and Library Construction

The total RNA of each sample was isolated with the RNeasy Plus Mini Kit (Qiagen, Germany) following the instructions of the manufacturer. Afterwards, RNA quality and concentration were determined using a NanoDrop 2000 (Thermo Scientific, DE, United States) and an Agilent Bioanalyzer 2100 (Agilent Technologies, CA, United States), respectively. Equal amounts of total RNA from each of nine sampling stages (excluding Ever 9) were pooled to generate one mixed sample for SMRT library preparation. Samples from Ever 9 were in a similar growth status with those from Ever 10, thus total RNA of Ever 9 were removed in order to include more interesting transcriptional changes in the transcriptome dataset under the premise of limited sequencing library capacity. First-strand cDNA was synthesized using a SMARTer PCR cDNA Synthesis Kit (Clontech, CA, United States). After a few rounds of PCR amplification, the amplified cDNA was divided into different size fractions to prevent preferential small template sequencing using a Blue Pippin (Sage Science, MA, United States). Three Iso-seq libraries (1–2 kb, 2–3 kb, and 3–6 kb) were then constructed and sequenced on three, two and 2 cells with the Pacific Bioscience RS II platform (PacBio, CA, United States), respectively.

**FIGURE1:**
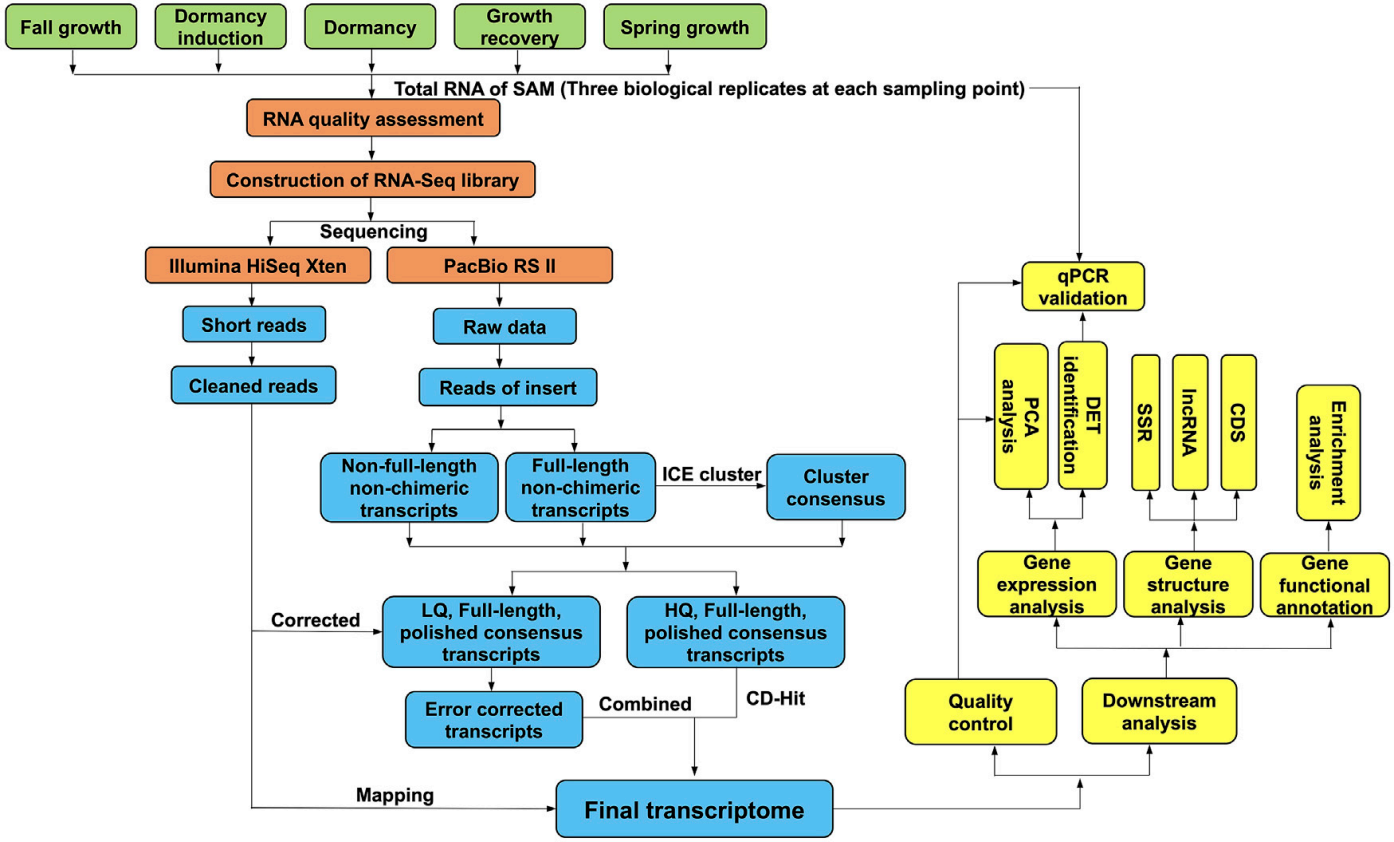
Overview of the experimental design and analysis pipeline. The RNA samples of Japanese iris from five crucial developmental stages related to winter dormancy were sequenced both by Pacbio Iso-seq and Illumina short read RNA-seq. The raw data from a Pacbio RS II sequencer was classified and corrected to generate the high-quality full-length transcripts (FLTs). The FLTs were used for gene annotation and structure analysis. Then the Illumina clean data were mapped to the FLTs to calculate the read counts of each transcript by RSEM and used to identify the differentially expressed transcripts with DESeq R package. The sample stage was determined by the observation of shoot growth or dormancy status.

Then, the above samples from five developmental stages essential for research on winter dormancy were selected for NGS sequencing according to their shoot growth or dormancy status. These five stages were fall growth (FG), dormancy induction (DI), dormancy (D), growth recovery (GR) and spring growth (SG), which corresponded to Ever 1, Ever 2, Ever 7, Ever 8 and Ever 10, respectively (Figure 1). Three biological replicates of each group were used to construct cDNA libraries following the Illumina standard operating procedure. Libraries were sequenced on an Illumina Xten platform (Illumina, CA, United States) to generate paired-end reads. The high-throughput sequencing (both SMRT and NGS) reported in this study was performed by Biomarker Technologies (Beijing, China).

### “SMRT + NGS” RNA-Seq Strategy and Analysis Workflow

The SMRT subreads were filtered using the standard protocols in the SMRT Analysis software package v2.3.0 (https://smrt-analysis.readthedocs.io/en/latest/SMRT-Pipe-Reference-Guide-v2.3.0/), and reads of insert (ROIs) were obtained using the Iso-seq pipeline with minFullPass = 0 and minPredictedAccuracy = 0.90. After examining the poly(A) signal and 5′ and 3′ adapters, FL and non-full-length (NFL) cDNA reads were recognized. Subsequently, consensus isoforms were identified using the algorithm of iterative clustering for error (ICE) correction and further polished to obtain high-quality consensus isoforms. The raw NGS short reads were filtered to remove adapter sequences, ambiguous reads with “N” bases, and low-quality reads. Afterwards, error correction of low-quality isoforms was conducted using the NGS reads with the proovread v2.13.13 software (Hackl et al., 2014). Redundant isoforms were then removed to generate a high-quality transcript dataset using the program CD-HIT v4.6.142 (Li and Godzik, 2006) with an identity threshold of 0.99 for the subsequent analysis.

### Quality Validation, Completeness Evaluation and Data Normalization

First, clean NGS data were obtained by removing low-quality reads and those containing adapters or poly-N and raw data in fastq format. Moreover, we applied FastQC v0.11.6 (Andrews, 2010) to assess the quality of the clean reads, including the mean per sequence quality scores, per base quality scores, and GC contents. A benchmarking universal single-copy orthologs (BUSCO) v4.0.2 (Simão et al., 2015) assessment was used to estimate the completeness of the SMRT transcriptomic sequencing. The completeness of the datasets in the processing steps, including high-quality and corrected low-quality polished consensus data, and the final FL transcript data were analysed in this study.

The clean NGS reads were then mapped to the FL transcriptome by Bowtie 2 (Langmead and Salzberg, 2012) using the end-to-end and sensitive modes. The read counts of each transcript were calculated using RNA-seq by Expectation Maximization (RSEM) (Li and Dewey, 2011), and the expected number of fragments per kilobase of transcript sequence per million base pairs (FPKM) was calculated to assess the expression levels.

### Downstream Analyses

Functional annotations were conducted by using the BLAST toolkit v2.2.26 (McGinnis and Madden, 2004) (E-value cut off 1e−5) against different protein and nucleotide databases including Clusters of Orthologous Groups (COG); evolutionary genealogy of genes: Non-supervised Orthologous Groups (eggNOG); euKaryotic Orthologous Groups (KOG); Kyoto Encyclopedia of Genes and Genomes (KEGG); Pfam, a database of conserved Protein families or domains; Swiss-Prot, a manually annotated, nonredundant protein database; Nr, the National Center for Biotechnology Information (NCBI) nonredundant protein database; and Gene Ontology (GO). Afterwards, a classification of the FL transcripts was conducted based on the COG annotation.

Simple sequence repeats (SSRs) in transcripts ≥500 bp in size were identified using the MIcroSAtellite identification tool (MISA). To predict the open reading frames (ORFs) in the transcripts, the package TransDecoder v3.0.0 (http://transdecoder.sf.net) was applied to define putative coding sequences (CDSs). The predicted CDSs were confirmed by blast against the protein databases Nr, Swiss-Prot and Pfam to identify transcripts containing complete ORFs. Furthermore, we screened all the transcripts for putative long non-coding RNAs (lncRNAs) using four computational approaches, including coding-non-coding index (Sun et al., 2013) (CNCI, score <0), coding potential calculator (Kong et al., 2007) (CPC, score <0), coding potential assessment tool (Wang et al., 2013) (CPAT), and the Pfam database to identify non-protein-coding RNA candidates and putative protein-coding RNAs from the unknown transcripts. The transcripts detected by all these programs were considered high-confidence lncRNAs (Yin et al., 2019).

A differential expression analysis between every two adjacent groups was performed using the DESeq R package v1.10.1 (Anders and Huber, 2010). The resulting *p* values were adjusted using Benjamini and Hochberg’s approach for controlling the false discovery rate (FDR). FL transcripts identified by DESeq with FDR ≤0.01 and fold change ≥2 were defined as differentially expressed. For visualization, volcano plots were drawn with TBtools v0.6695 (Chen et al., 2020), and a Venn diagram was drawn using an online tool developed by Ugent University (http://bioinformatics.psb.ugent.be/webtools/Venn/). GO enrichment analysis was performed using topGO (Alexa and Rahnenfuhrer, 2010) with Fisher’s exact test based on GO categories including biological process, cellular component and molecular function, and KOBAS software (Xie et al., 2011) was used to test the statistical enrichment of KEGG pathways among differentially expressed transcripts (DETs) between every two adjacent developmental groups of Japanese iris. To detect the dynamic patterns of gene expression, the k-means clustering analysis of all DETs was performed on samples from five crucial developmental stages using BMKcloud online tools (http://www.biocloud.net).

### Sample Reproducibility and Real-Time qPCR Validation

Principal component analysis (PCA) was applied to assess the sample reproducibility among the three biological replicates of each group based on the FPKM values of each transcript. Additionally, the classification of the samples shown in the PCA could further confirm the developmental or dormancy status of each sample, which was noted at sampling. The PCA plot was produced by the online tools at Biomarker Technologies (http://www.biocloud.net/).

To confirm the results of the gene expression analysis, the extracted total RNA of the samples used for NGS was also used for quantitative real-time polymerase chain reaction (RT-qPCR) validation. Beacon Designer v7.7 (PREMIER Biosoft, CA,United States) was used to design gene-specific primers for qRT-PCR. Then, qRT-PCR was performed with SYBR^®^ Premix Ex Taq (TaKaRa, Japan) on a CFX Connect™ Real-Time PCR Detection system (Bio-Rad, CA, United States) under the following conditions: denaturation at 95°C for 2 min; 39 cycles of 95°C for 5 s and 55°C for 30 s; and a melting curve program of 95°C for 5 s, 65°C for 5 s and 95°C for 5 s. Nine DETs were selected randomly for qRT-PCR validation and normalized against the reference gene *POLYPYRIMIDINE TRACT-BINDING PROTEIN 1* (*PTB1*) (Hao et al., 2014; Ma et al., 2016). The primers used to amplify these genes are shown in Supplementary Table S1. Relative gene expression levels were calculated using the 2^−ΔΔCt^ method (Li et al., 2019; Xu et al., 2019).

### Code Availability

**SMRT Analysis software package v2.3.0:** http://www.pacb.com/products-and- services/analytical-software/smrt-analysis/.
**proovread v2.13.13:** https://github.com/BioInf-Wuerzburg/proovread/.
**CD-HIT v4.6.142:** http://weizhongli-lab.org/cd-hit/.
**FastQC v0.11.6:** https://www.bioinformatics.babraham.ac.uk/projects/fastqc/.
**BUSCO v4.0.2:** https://busco.ezlab.org/.
**Bowtie2:** https://sourceforge.net/projects/bowtie-bio/files/bowtie2/.
**RSEM v1.1.17:** http://deweylab.github.io/RSEM/.
**BLAST toolkit v2.2.26:** https://www.ncbi.nlm.nih.gov/IEB/ToolBox/CPP_DOC/doxyhtml/group__AlgoBlast.html/.
**topGO package:** http://www.bioconductor.org/packages/release/bioc/vignettes/topGO/inst/doc/topGO.R/.
**KOBAS v2.0:** http://kobas.cbi.pku.edu.cn/kobas3/.
**MIcroSAtellite identification tool:** http://pgrc.ipk-gatersleben.de/misa/http://pgrc.ipk-gatersleben.de/misa/.
**TransDecoder v3.0.0**: https://github.com/TransDecoder/TransDecoder/.
**CNCI:** https://github.com/www-bioinfo-org/CNCI/.
**CPC:** https://github.com/biocoder/cpc/.
**CPAT:** http://lilab.research.bcm.edu/cpat/.
**DESeq R package v1.10.1:** https://www.rdocumentation.org/packages/DESeq2/versions/1.12.3/topics/DESeq/.
**TBtools v0.6695:** https://github.com/CJ-Chen/TBtools/.

## Technical Validation

### RNA Quality Control

All the RNA samples used for NGS short-read RNA-seq library construction had 260:280 ratios between 1.8 and 2.3 and RNA integrity numbers (RINs) ≥ 6.5. To obtain high-quality reads for downstream analyses, the minimum RIN value of the RNA samples used for SMRT FL sequencing was 7.5 (Supplementary Table S2; Supplementary Table S3).

### RNA-Seq Quality Validation

For the clean short-read RNA-seq data, we used FastQC to verify whether the 142.13 Gb of data was suitable for hybrid error correction and gene expression analysis of the FL transcriptome. The results showed that over 93.1% of the per base quality scores were above 30, and the GC content ranged from 48.1 to 51.2% in all samples (Supplementary Table S2), indicating the high quality of the RNA-seq data.

A total of 10.57 Gb clean data obtained from the PacBio RS II platform were processed into 300,204 error-corrected ROIs with PacBio Iso-seq and then classified into 138,363 full-length non-chimeric (FLNC) reads with 5′ primer, 3′ primer and poly-A tail (Supplementary Table S4; Supplementary Figures S1A, B). Subsequently, 67,787 consensus isoforms were established using the ICE program in SMRT Analysis (Figure 1C). The high-quality consensus isoforms with an accuracy over 99% were selected by Quiver algorithm in the SMRT Analysis software package v2.3.0, and the low-quality consensus isoforms were corrected with the NGS data using proovread. Finally, 58,654 corrected FL isoforms were used for further analyses after removing redundant transcripts with CD-HIT. We applied BUSCO v4.0.2 to estimate the completeness of the FL transcripts and found that 81.2% of the transcripts were complete; this value was much higher than that of either the high-quality consensus transcripts (76.9%) or the corrected low-quality consensus transcripts (44.3%, Supplementary Figure S1D). All these analyses demonstrated that the sequencing data in this study were adequately complete and suitable for downstream analyses.

### Annotation Quality and Gene Structure Validation

To obtain comprehensive information on gene function in Japanese iris, all 58,654 FL transcripts were annotated with eight databases (Supplementary Figure S2A). A total of 56,742 (96.74%) transcripts were annotated in at least one database, of which the largest number (56,646; 96.57%) had similar sequences in Nr, and the smallest number (25,114; 42.82%) matched COG. Over half of the FL transcripts were annotated in the GO, KOG, Pfam, Swiss-Prot, eggNOG and Nr databases. With respect to the functional classifications by COG, most transcripts were designated “General function prediction only” (6,637), “Posttranslational modification, protein turnover, chaperones” (2,739), and “Carbohydrate transport and metabolism” (1,694; Supplementary Figure S2B).

SSR markers represent one of the most widely used molecular markers in numerous organisms. Here, 50,541 SSRs were found from transcripts over 500 bp, and most of them had perfect mono- (p1), di- (p2), or tri-(p3) nucleotide repeats (Supplementary Figure S2C). Moreover, lncRNAs constitute a major component of the transcriptome and have recently received increasing attention. We identified 1,015 candidate lncRNAs in Japanese iris with high confidence for the first time in this study (Supplementary Figure S2D). The length distribution of the predicted CDSs containing complete ORFs is shown in Supplementary Figure S2E. The above gene structure information of Japanese iris derived from this high-quality FL transcriptome could benefit further research in the *Iris* genus, for which no genomic information is available as yet.

### Gene Expression and RT-qPCR Validation

**FIGURE 2:**
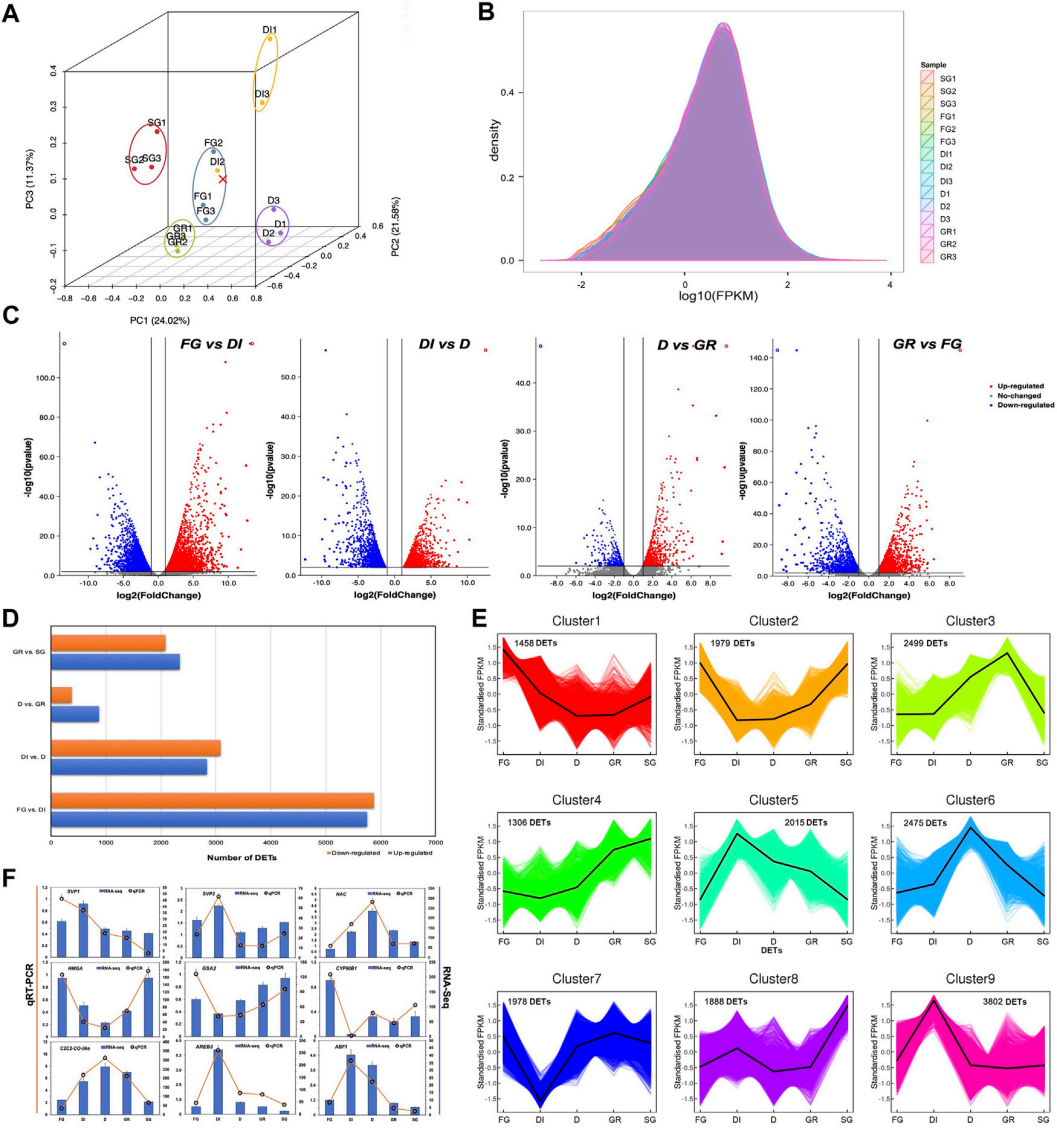
Quality assessment and comparisons of transcriptome data between every two adjacent developmental groups of Japanese iris. **(A)** The principal component analysis (PCA) plot of the samples. The plot depicts the clustering patterns of the samples according to the developmental stages related to winter dormancy. In the diagram, the *x*-, *y*- and *z*-axis represent the first, second and third principal components. The different colors represent the developmental stages of sampling. FG represents fall growth. DI represents dormancy induction. D represents dormancy. GR represents growth recovery. SG represents spring growth. **(B)** The gene expression density of the samples. The *x*-axis represents log_10_(FPKM) in each sample. The *y*-axis represents the density of gene expression in each sample. **(C)** Distribution of differentially expressed transcripts (DETs) between every two adjacent developmental groups of Japanese iris. The downregulated and upregulated DETs are high-lighted in blue and red, respectively. **(D)** The number of DETs between every two adjacent developmental groups. The *x*-axis represents the number of DETs. The *y*-axis represents the four pairs of comparisons. **(E)** Dynamic gene expression patterns of all DETs by the k-means clustering analysis. The *x*-axis represents the five developmental stages of sampling. The *y*-axis represents the normalized gene expression from RNA-Seq. **(F)** Validation of differential expression of nine genes from real time qPCR (RT-qPCR) and RNA-Seq. The *x*-axis represents the five developmental stages of sampling. The left *y*-axis represents the gene expression from RT-qPCR and the right *y*-axis represents the gene expression from RNA-Seq.

The correlation of gene expression levels among samples is an important index to verify the reliability of an experiment. The PCA of the RNA-seq data revealed a high correlation among all the samples except DI2 (Figure 2A). This result was probably caused by the complexity of the inner physiological and biochemical changes in each sample. Thus, the gene expression data of DI2 were removed for subsequent expression research. Figure 2B shows the density of the population distribution for the gene expression levels in each sample. Additionally, we performed pairwise analyses of the expression profiles of the adjacent developmental groups (FG vs. DI, DI vs. D, D vs. GR, GR vs. SG) and identified a total of 16,061 DETs with the parameters fold change ≥2 and FDR value <0.01 using DESeq. The volcano plot shows downregulated and upregulated DETs highlighted in blue and red, respectively (Figure 2C). Notably, the largest number of DETs was identified between FG and DI, indicating dramatic changes during the process of DI (Figure 2D). To identify DETs with co-expressed patterns, the k-means clustering analysis was applied based on the inputs of normalized FPKM values of all DETs. (Figure 2E). The results indicated that all DETs could be divided into nine clusters with distinct gene expression patterns. Of these clusters, cluster 9 contained the largest number of 3,802 DETs with obvious upregulation only in DI, which was highly consistent with the above DET analysis. Furthermore, numbers of DETs in cluster 3, cluster 6, and cluster 5 were over 2,000. DETs in those clusters exhibiting distinct gene expression at DI, D, and GR stages, should be paid attention as well.

Gene enrichment analysis can classify transcripts into different categories according to functional annotations and reveal the critical pathways involved in a specific biological process. In this study, a total of 38,563 transcripts were annotated to multiple GO classification terms. GO and KEGG enrichment of DETs between every two adjacent developmental groups of Japanese iris, including FG vs DI, DI vs D, D vs GR, and GR vs SG were analysed in details. As for GO enrichment in biological process category, the most significantly enriched GO terms were “Oxidation-reduction process” during the whole experiment (Supplementary Figure S3A). Besides, the majority of DETs were enriched in GO terms, such as “DNA replication”, “Photosynthetic electron transport in photosystem I”, and “Chlorophyll biosynthetic process”. On the other hand, the KEGG enrichment results indicated that these DETs related to stage conversion were significantly enriched in pathways, including “Starch and sucrose metabolism”, “DNA replication”, and “Phenylalanine metabolism or biosynthesis” (Supplementary Figure S3B). Although the log2 fold change values from the transcriptomic analysis and qPCR analysis were different, the differential expression levels of these nine randomly selected DETs by RT-qPCR were highly consistent with those observed by RNA-Seq (Figure 2F), confirming the high accuracy of the RNA sequencing and analyses in this experiment.

## Conclusion

In summary, the data provided in this study represent a high-quality FL transcriptomic dataset characterizing winter dormancy in Japanese iris for the first time. Additionally, we screened multiple DETs associated with the growth and winter dormancy transition of Japanese iris, which may play crucial roles in this process. This dataset provides a valuable resource for gene annotation, structure and functional analysis in the genus *Iris* and supports a better understanding of the biological process of winter dormancy and foliar habits in evergreen herbaceous perennials, especially monocotyledons.

## Data Availability

The clean data of this study were deposited into the NCBI Sequence Read Archive (SRA) under the the BioProject PRJNA486414. The biosample accession numbers for the NGS and SMRT RNA-seq data are listed in Supplementary Tables S2, S3, respectively. The results of the functional annotation, gene structure, and DET analyses are available in Figshare (https://doi.org/10.6084/m9.figshare.19159484.v2). The above information was publicly available.
