# Supplementary material for: Hybrid RNA Sequencing Strategy for the Dynamic Transcriptomes of Winter Dormancy in an Evergreen Herbaceous Perennial, Iris japonica
Source: Front Genet. 2022 Mar 16;13:841957. doi: 10.3389/fgene.2022.841957 (PMC8965894; doi:10.3389/fgene.2022.841957)
Supplement: Supplementary file 3 [file Presentation1.PDF]

Supplementary Material

The Supplementary Material for this article can be found online at:

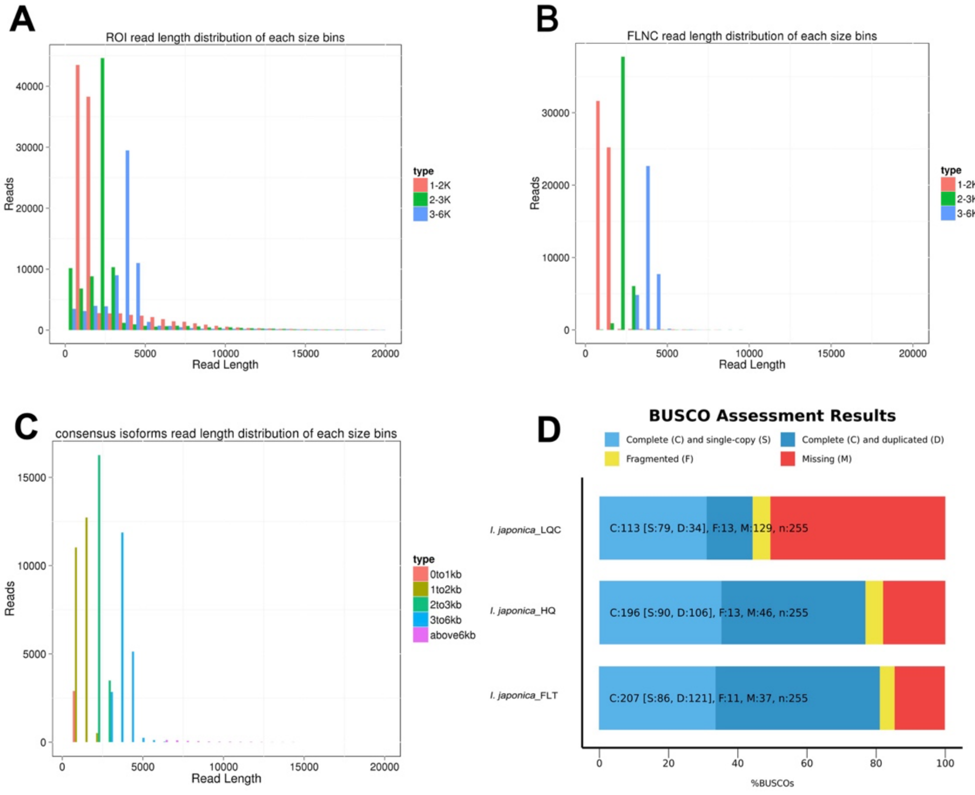

Supplementary Figure 1. The statistic results and completeness assessment of the transcripts in single molecular real time (SMRT) sequencing. **(A)** Error corrected reads of insert (ROI) read length distribution of each size bins. The *x*-axis represents ROI read length. The *y*-axis represents the number of ROI reads with specific lengths. **(B)** Full-length, non-chemiric (FLNC) read length distribution of each size bins. The *x*-axis represents FLNC read length. The *y*-axis represents the number of FLNC reads with specific lengths. **(C)** Consensus isoforms read length distribution of each size bins. The *x*-axis represents consensus isoforms read length. The *y*-axis represents the number of consensus isoforms reads with specific lengths. **(D)** The completeness of transcripts in SMRT sequencing. The completeness of transcripts was assessed by benchmarking universal single-copy ortholog (BUSCO). The *x*-axis represents the percentage of detected BUSCOs. The light blue diamond represents complete (C) and single-copy (S) genes; the dark blue represents complete and duplicated (D) genes; the yellow diamond represents fragmented (F) genes; the red diamond represents the missing (M) genes. *I. japonica*_LQC represents corrected low-quality transcripts of Japanese iris in SMRT sequencing. *I. japonica*_HQ represents high-quality transcripts of Japanese iris in SMRT sequencing. *I. japonica*_FLT represents full-length transcripts of Japanese iris by combined SMRT and next generation sequencing.

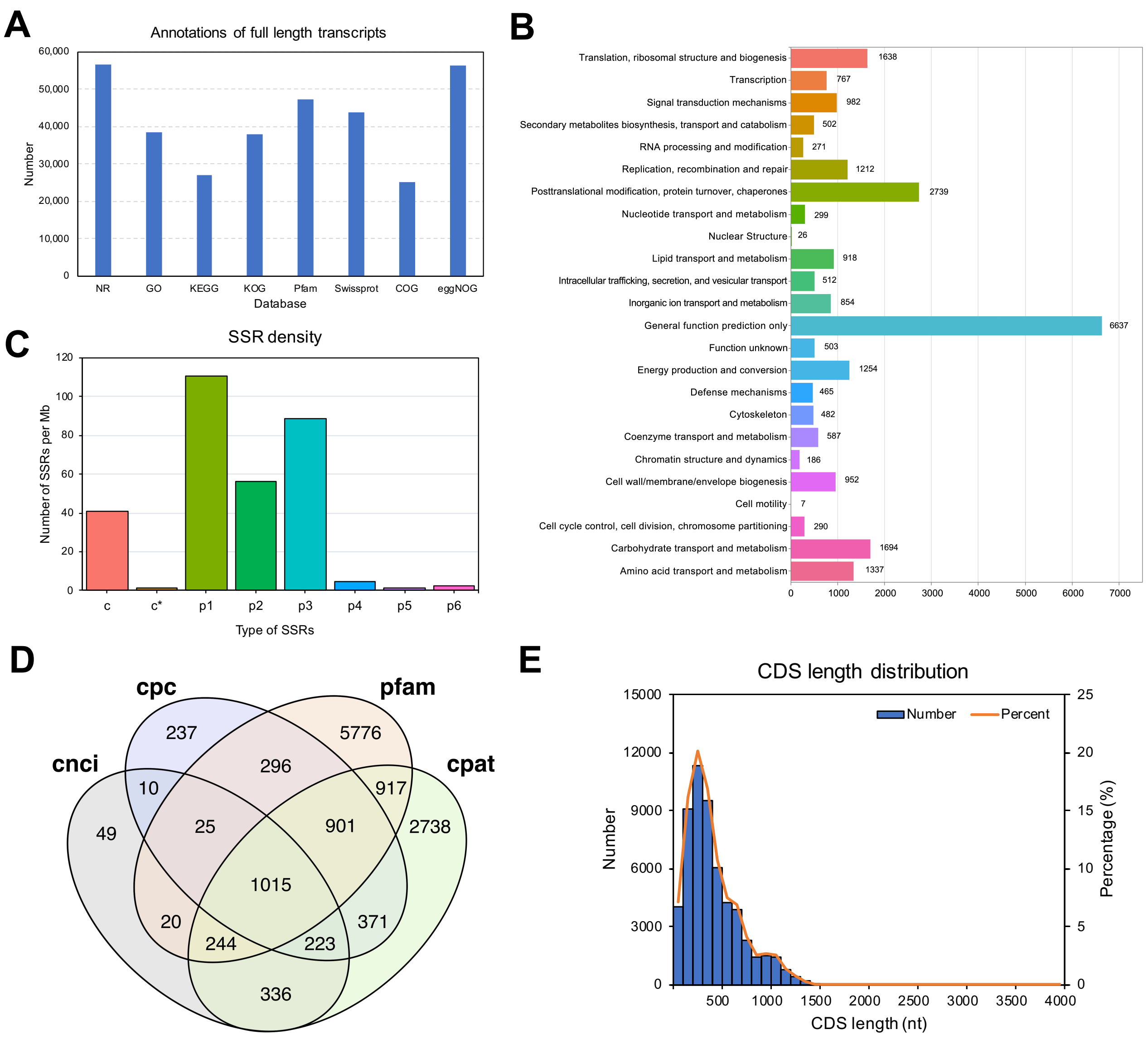

Supplementary Figure 2. The functional annotations and results of gene structure analyses of the full-length transcripts (FLTs) derived from the combined single molecular real time and next generation sequencing. **(A)** The FLTs were annotated in eight datasets. **(B)** COG classification of the FLTs. The *x*-axis represents the number of FLTs classified in each category. The *y*-axis represents the 24 categories of COG dataset where the FLTs classified. **(C)** The density and types of identified SSRs from the FLTs. The *x*-axis represents the types of SSRs. c and c* represent SSRs present in compound formation. p1 to p6 represent mono-, di-, tri-, tetra-, penta-, or hexa-nucleotide repeats, respectively. **(D)** Venn diagram of the number of identified lncRNA in four computational approaches, including CNCI, CPC, Pfam and CPAT. **(E)** The length distribution of predicted CDS containing complete ORFs using TransDecoder. The *x*-axis represents the CDS length. The left *y*-axis represents the number of CDS with specific lengths. The right *y*-axis represents the percentage of CDS with specific lengths.

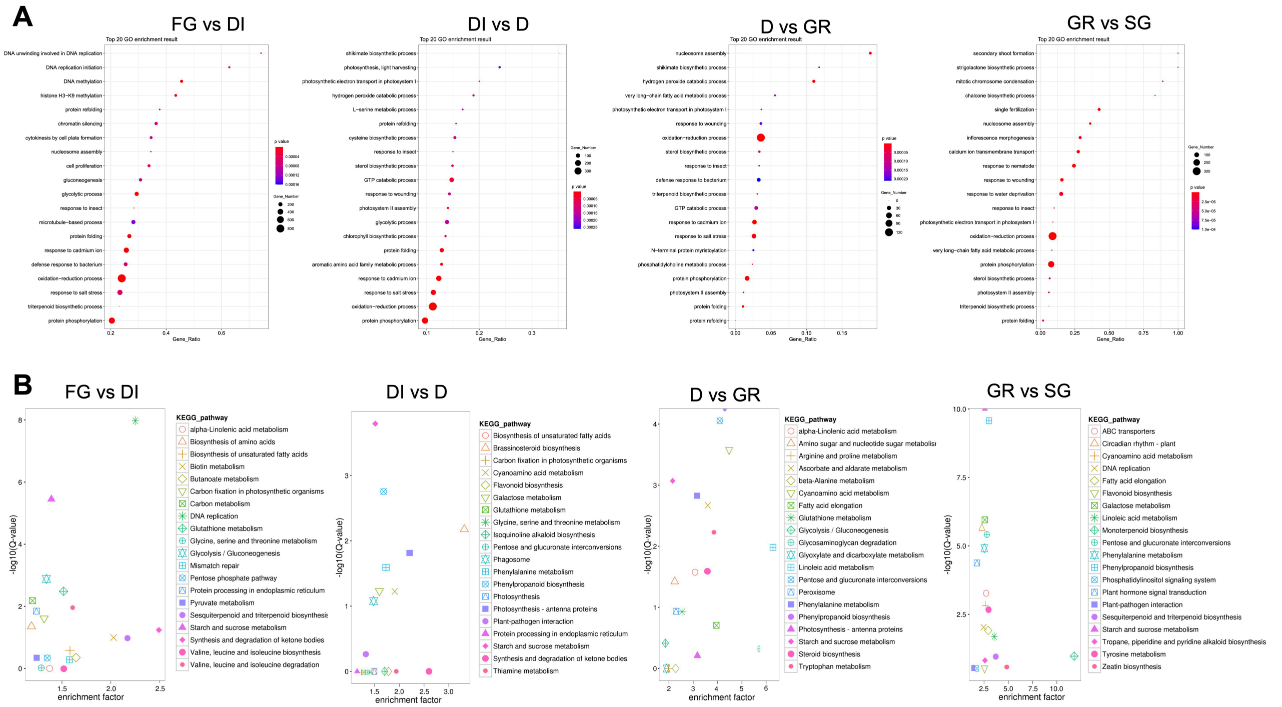

Supplementary Figure 3. The GO and KEGG enrichment analyses of differentially expressed transcripts (DETs) between every two adjacent developmental groups of Japanese iris. (A) The top 20 enriched GO terms in biological process obtained by DETs between fall growth (FG) vs dormancy induction (DI), DI vs dormancy (D), D vs growth recovery (GR), and GR vs spring growth (SG), respectively. (B) The top 20 enriched KEGG pathways obtained by DETs between FG vs DI, DI vs D, D vs GR, and GR vs SG, respectively.
